# Molecular HLA mismatching for prediction of primary humoral alloimmunity and graft function deterioration in paediatric kidney transplantation

**DOI:** 10.3389/fimmu.2023.1092335

**Published:** 2023-03-15

**Authors:** Jon Jin Kim, Alexander Fichtner, Hannah C. Copley, Loren Gragert, Caner Süsal, Luca Dello Strologo, Jun Oh, Lars Pape, Lutz T. Weber, Marcus Weitz, Jens König, Kai Krupka, Burkhard Tönshoff, Vasilis Kosmoliaptsis

**Affiliations:** ^1^ Department of Surgery, University of Cambridge, Cambridge, United Kingdom; ^2^ Department of Paediatric Nephrology, Nottingham University Hospital, Nottingham, United Kingdom; ^3^ Department of Pediatrics I, University Children’s Hospital Heidelberg, Heidelberg, Germany; ^4^ School of Medicine, Tulane University, New Orleans, LA, United States; ^5^ Transplantation Immunology, Institute of Immunology, University Hospital Heidelberg, Heidelberg, Germany; ^6^ Renal Transplant Unit Bambino Gesù, Children’s Hospital, Rome, Italy; ^7^ University Hospital Hamburg, Pediatric Nephrology, Hamburg, Germany; ^8^ Clinic for Paediatrics III, Essen University Hospital, Essen, Germany; ^9^ Pediatric Nephrology, Children’s and Adolescents’ Hospital, University Hospital Cologne, Cologne, Germany; ^10^ University Hospital Tübingen, Pediatric Nephrology, Tübingen, Germany; ^11^ Department of General Pediatrics, University Children’s Hospital, Münster, Germany; ^12^ NIHR Blood and Transplant Research Unit in Organ Donation and Transplantation at the University of Cambridge and the NIHR Cambridge Biomedical Research Centre, Cambridge, United Kingdom

**Keywords:** kidney transplantation, HLA mismatch, molecular mismatch, eplets, predictive modeling, antibody mediated rejection (ABMR), donor specific HLA antibodies

## Abstract

**Introduction:**

Rejection remains the main cause of allograft failure in paediatric kidney transplantation and is driven by donor-recipient HLA mismatching. Modern computational algorithms enable assessment of HLA mismatch immunogenicity at the molecular level (molecular-mismatch, molMM). Whilst molMM has been shown to correlate with alloimmune outcomes, evidence demonstrating improved prediction performance against traditional antigen mismatching (antMM) is lacking.

**Methods:**

We analysed 177 patients from the CERTAIN registry (median follow-up 4.5 years). molMM scores included Amino-Acid-Mismatch-Score (AAMS), Electrostatic-Mismatch-Score (EMS3D) and netMHCIIpan (netMHC1k: peptide binding affinity ≤1000 nM; netMHC: binding affinity ≤500 nM plus rank <2%). We stratified patients into high/low-risk groups based on risk models of DSA development.

**Results:**

Donor-specific HLA antibodies (DSA) predominantly targeted the highest scoring molMM donor antigen within each HLA locus. MolMM scores offered superior discrimination versus antMM in predicting *de novo* DSA for all HLA loci; the EMS3D algorithm had particularly consistent performance (area under the receiver operating characteristic curve (AUC) >0.7 for all HLA loci vs. 0.52-0.70 for antMM). ABMR (but not TCMR) was associated with HLA-DQ molMM scores (AAMS, EMS3D and netMHC). Patients with high-risk HLA-DQ molMM had increased risk of graft function deterioration (50% reduction in baseline eGFR (eGFR50), adjusted HR: 3.5, 95% CI 1.6-8.2 high vs. low EMS3D). Multivariable modelling of the eGFR50 outcome using EMS3D HLA-DQ stratification showed better discrimination (AUC EMS3D vs. antMM at 2 years: 0.81 vs. 0.77, at 4.5 years: 0.72 vs. 0.64) and stratified more patients into the low-risk group, compared to traditional antMM.

**Conclusion:**

Molecular mismatching was superior to antigen mismatching in predicting humoral alloimmunity. Molecular HLA-DQ mismatching appears to be a significant prognostic factor for graft function deterioration in paediatric kidney transplantation.

## Introduction

Kidney transplantation is the optimal treatment for paediatric patients with kidney failure conferring a survival advantage and superior quality of life compared to dialysis. Nevertheless, the median transplant life-span has remained approximately 12-15 years and most paediatric patients require more than one transplant in their lifetime (1). Human leukocyte antigen (HLA) mismatching is of prime importance in determining long-term allograft survival and subsequent HLA sensitisation, restricting access to re-transplantation in patients whose primary graft has failed (1–4). In turn, increased sensitisation leads to prolonged time on dialysis with an associated five-fold increased risk of death (5).

Current methods for histocompatibility assessment simply enumerate the number of HLA mismatches, defined at the serological level, and are predicated on the assumption that all mismatches within a locus are of equal significance (6). Recent advances in HLA sequencing technology and availability of computational approaches for studying B- and T-cell epitopes have led to the development of algorithms that aim to determine the degree of donor-recipient incompatibility at the molecular level (‘molecular HLA mismatch’, molMM). Accordingly, HLA incompatibility can be defined at the amino acid sequence level either by enumerating amino acid mismatches (AAMS) on donor compared to recipient HLA (Cambridge HLA immunogenicity algorithm (7, 8)) or by enumerating small patches of polymorphic amino acids at or near the molecular surface of HLA (termed ‘eplets’), as implemented in the HLAMatchmaker program (9). The sum of donor HLA sequence polymorphisms (amino acid load or eplet load) is thus used to determine donor HLA immunogenicity. Many studies have used the eplet load to predict the risk of rejection, development of donor-specific HLA antibodies (DSA), and graft failure (10–14). Despite good correlation between donor HLA total eplet load and humoral alloresponses, DSA may develop against HLA with low eplet mismatches and vice versa, and it is important to consider additional factors such as immunosuppression levels and patient adherence with medication (15–18). Eplets have also been proposed as the structural basis of B-cell epitopes that may be confirmed experimentally (‘antibody-verified’ eplets), although the evidence for the validity and utility of this approach remains weak (12, 18, 19).. Alternatively, HLA molecular mismatch can be defined at the structural level using a computational algorithm to quantify surface electrostatic potential differences between HLA molecules at the tertiary level (electrostatic mismatch score three dimensional or EMS3D) (6, 20–22). This algorithm is theoretically appealing, considering the structural nature of B-cell receptor recognition; however, it is not yet clear whether the EMS3D approach offers a superior assessment of HLA incompatibility compared to amino acid sequence methods alone. Finally, it is important to emphasise that adaptive alloimmune responses depend on recognition of donor HLA-derived peptides, presented in the context of recipient HLA class II, by CD4^+^ T-cells. NetMHCIIpan is arguably the most widely used computational approach for identifying putative CD4^+^ T-cell epitopes, based on prediction of peptide binding affinity by HLA class II molecules. NetMHCIIpan was trained on experimental data using a neural network method (23, 24). A previous version of this method (v.3.0) has been implemented into the PIRCHE program (predicted indirectly recognisable HLA epitopes) (25, 26). Most of the studies using PIRCHE in transplantation defined putative donor HLA-derived peptide epitopes using only recipient HLA-DR as presenting molecules utilising a low affinity binding threshold of <1000 nM.

In this paediatric kidney transplantation study, we aimed to assess modern molecular methods for determining HLA incompatibility in addition to the currently used HLA antigen mismatch (antMM), defined at the serological split resolution level. For HLA molMM methods, we utilised the AAMS, EMS3D and NetMHCIIpan algorithms. Specifically for NetMHCIIpan, we incorporated recipient HLA-DRB1, DRB3/4/5 and -DQ molecules for assessment of peptide presentation and examined two methods for selecting HLA class II peptide binders: a stringent method using peptide binding affinity threshold of ≤500 nM plus binding affinity rank <2% (denoted as netMHC in this manuscript) and another method using a peptide affinity threshold of ≤1000 nM, akin to the PIRCHE-II approach (denoted as netMHC1k) (27). We examined the ability of these methods to predict allo-immune outcomes and risk of graft dysfunction in a multi-centre cohort of paediatric kidney transplant recipients. We show that molMM methods were superior to antMM in predicting post-transplant humoral alloreactivity. Moreover, molMM methods, as exemplified by the EMS3D algorithm, had superior performance in stratifying patients according to risk of graft function deterioration compared to conventional HLA antigen mismatching.

## Results

### Population characteristics

A total of 177 patients from nine centres were included utilising a cohort of the Cooperative European Paediatric Renal Transplant Initiative (CERTAIN) registry (**Table 1**). Patients were representative of a paediatric transplant population: median age 10.8 years (IQR 4.9-14.7), of which 60% were male and 40% had underlying congenital abnormalities of the kidney and urinary tract. Patients represented a low HLA sensitisation group: 82% of patients had panel reactive antibody reactivity of 0%, and 92% were first transplants. 64 (36%) patients had induction treatment with basiliximab and the majority had steroid and calcineurin inhibitor-based maintenance immunosuppression (172, 97%). Median follow-up time was 4.5 (IQR, 3.0-5.0) years. There were two patient deaths at 4.0 and 4.5 years post-transplant due to sepsis, and five allograft failures at 1.5, 2, 3.5, 4.5 and 5 years post-transplant.

**Table 1:** Patient demographics and characteristics.

Characteristics	Number (total n = 177)
Age (years)	10.8 (4.9 – 14.7)
HLA antigen mismatch	3 (2-3)
Male/Female	107 (60%)/70 (40%)
Ethnicity Caucasian Non-caucasian	168 (95%) 9 (5%)
Pre-emptive Transplant	46 (26%)
Primary Kidney Disease CAKUT Glomerular Other	72 (40%) 53 (30%) 52 (30%)
Living related/Deceased donor	67 (38%)/110 (62%)
Panel Reactive Antibodies (0%, 1-10%, 11-80%, 81-100%)	146 (82%), 20 (11%), 7 (4%), 4 (2%)
Graft number >1	14 (8%)
Immunosuppressive therapy Basiliximab induction Month 3: Steroids/CNI/MMF Steroids/CNI/mTOR Steroids/CNI/Azathioprine Steroids/CNI Steroid free CNI Intra-patient variability* ≤25%	64 (36%) 133 (75%) 22 (12%) 6 (3%) 10 (6%) 5 (3%) 120 (68%)

CAKUT, congenital abnormalities of the kidney and urinary tract; CNI, calcineurin inhibitor; MMF, mycophenolate mofetil; mTOR, mammalian target of rapamycin inhibitor, *calculated using mean absolute deviation.

Results are presented as median (inter-quartile range) and percentages.

### Correlation between molMM scores

**Figure 1:**
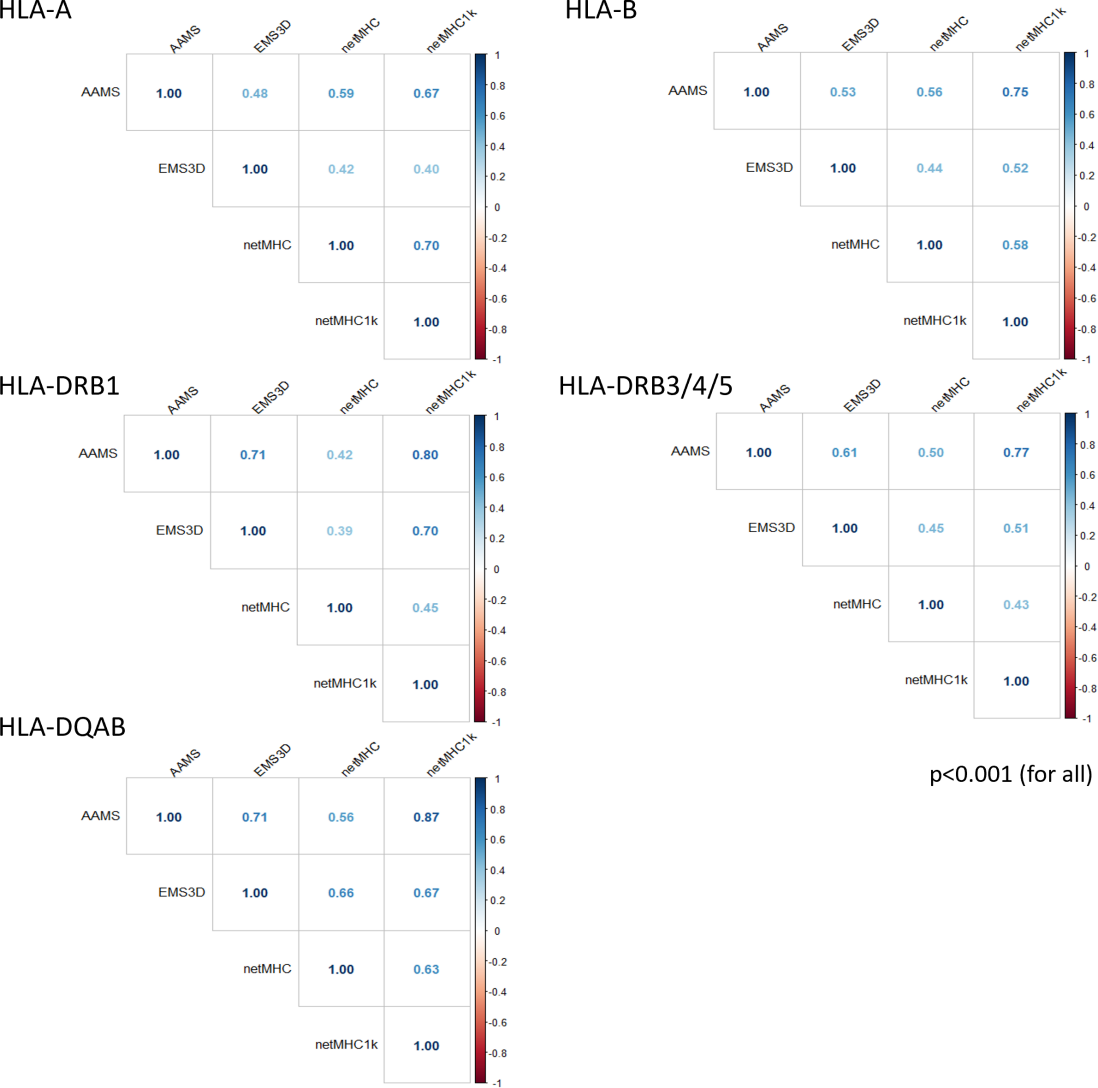
Correlation plots for the molecular HLA mismatch scores for each HLA locus. Results are shown using Pearson correlation (p-values <0.05 for all correlations).

As shown in **Figure 1**, there was wide variability in the degree of score correlation among molMM methods. Correlation with amino acid sequence polymorphism (as reflected in the AAMS) was highest with the netMHC1k score across all HLA loci examined (r=0.67-0.87, p<0.001), reflecting the low affinity threshold used in the latter method to select polymorphic donor HLA peptides as putative CD4^+^ T-cell epitopes. In contrast, correlation between AAMS and netMHC was poor (r=0.42-0.59, p<0.001) due to the stringent criteria used in the latter method to select HLA class II peptide binders. There was poor to moderate correlation between AAMS and EMS3D for HLA class I (r=0.48-0.53, p<0.001) and class II (r=0.61-0.71, p<0.001) loci respectively, consistent with the structural nature of the EMS3D score. Overall, the correlation among different molMM scores was highest for HLA class II comparisons.

### *De novo* donor-specific HLA antibody development

*De novo* DSA were detected in 56/177 (32%) patients: 9/56 (14%) class I only, 29/56 (52%) class II only, and 18/56 (32%) to both class I and class II. DSA incidence was highest against HLA-DQ (30/56, 54% of DSA positive patients) (**Table S1**).

For each patient, we first considered each donor mismatched HLA allele individually and used the single HLA molecule method to assess the ability of molMM scores to predict DSA development (10). Model discrimination was assessed using area under receiver operating characteristic (ROC) curves (AUC, **Figure S1**). For transplant pairs with two allelic mismatches at a particular locus, this method utilizes the highest scoring mismatch for risk stratification at the patient level. **Table S2** shows the comparison between antMM and molMM scores in predicting locus-specific DSA development at the patient level. Other than for HLA-A DSA, molMM methods had significantly better discrimination in their association with locus-specific DSA compared to antMM. Overall, EMS3D was the most consistent method for DSA prediction with AUCs >0.7 for all loci examined.

**Figure 2:**
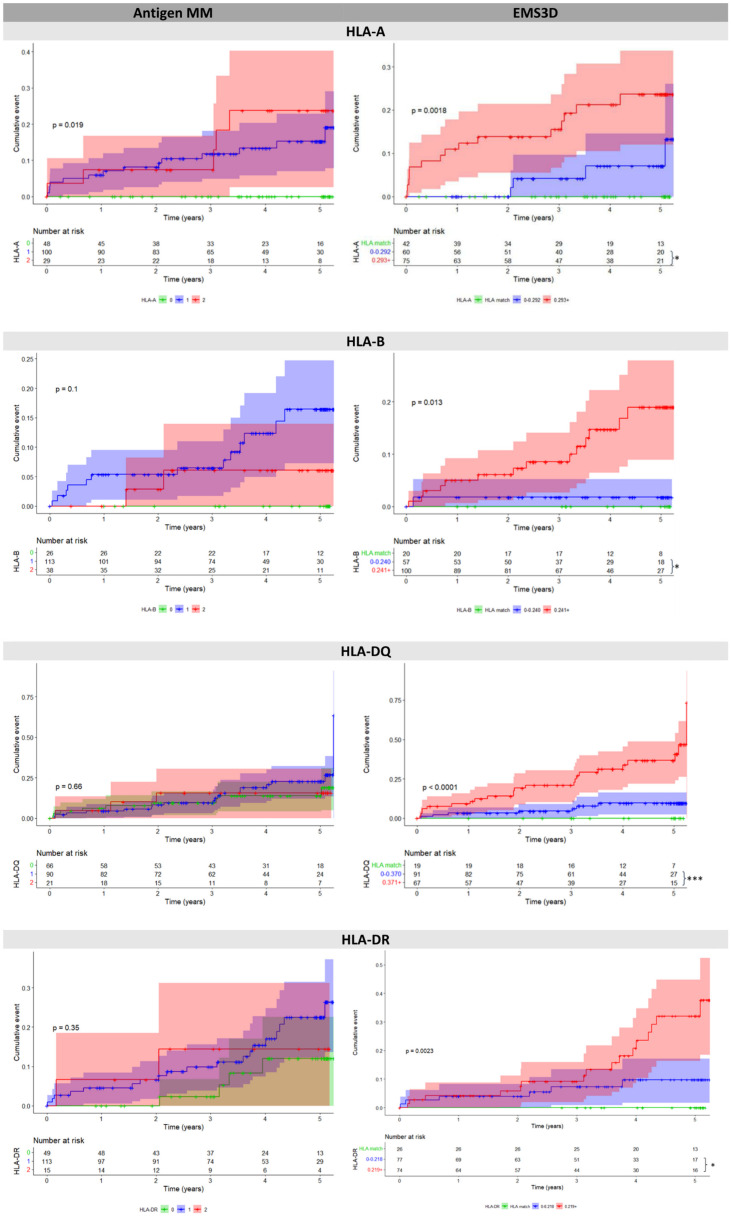
Cumulative event plots for *de novo* DSA at each HLA locus, comparing serological mismatching (left) versus EMS3D risk thresholds (right). For EMS3D, patients are categorised as HLA allelic match (green), low risk (≤Youden Index, blue) and high risk (>Youden Index, red). The Youden Index was calculated using the single molecule method (see methods) as shown in **Table S3**. Log-rank results comparing all patient groups in the model are shown in the graph. Log-rank analysis was performed comparing patients in the high risk versus the low risk group (HLA matched excluded). The shaded area depicts 95% confidence intervals for each curve. *p < 0.05 ***p < 0.001.

Our principal aim was to stratify patients according to risk of DSA development at a specific HLA locus, rather than simply establish the association between HLA incompatibility and alloantibody responses. We therefore used the Youden index from the single molecule analysis (**Table S3**) and categorised patients as HLA matched, those with low risk HLA mismatches (molMM score <Youden index) and patients with high risk HLA mismatches (molMM ≥Youden index) at a particular locus. **Figure 2** presents the results of analyses with DSA as a time dependent variable. For simplicity, and because of its discriminative ability in ROC curve analyses above, patient risk stratification according to EMS3D versus antMM is depicted (similar analyses for other molMM methods are shown in **Figure S2**). Overall, there was a significant association between HLA risk strata, as determined by EMS3D, and risk of DSA development, whereas this was not the case using traditional antigen mismatching, other than for risk strata within the HLA-A locus. More importantly, the incidence of DSA increased significantly in high compared to low EMS3D risk groups for all HLA loci (HLA-A: HR 3.2, 95% CI: 1.1-9.7, high vs. low risk, p<0.05 log rank test; HLA-B: 8.0, 95% CI 1.1-61, p<0.05; HLA-DQ: 5.0, 95% CI 1.2-11.7, p<0.001 and HLA-DR: 2.8, 95% CI 1.1-7.4, p<0.05). In contrast, the cumulative incidence of DSA development was not significantly different in patients with one versus two antigen mismatches for all HLA loci examined. Stratification using AAMS, netMHC and netMHC1k was more variable (**Figure S2**). Only AAMS stratification showed a significant difference in cumulative DSA incidence against HLA-B mismatches between low and high risk groups (but essentially stratified the majority of patients in the high risk group, n=121), whereas high versus low risk strata were not significantly different for DSA against HLA-A or -B using the other methods. For HLA-DQ mismatches, all molMM methods showed a significant difference in DSA free survival between high versus low risk patient groups. Finally, for HLA-DR mismatches, high versus low risk group comparisons were significantly different for AAMS and netMHC molMM scores, but not for netMHC1k. To exclude a confounding effect of immunosuppression levels on the supp associations, we examined how often patients with and without DSA had CNI levels above 5ng/ml (**Figure S3A**) and found no significant difference between the two groups (other than for high HLA-B risk patients where those with DSA were more likely to have CNI levels above 5ng/ml). We also compared CNI levels in the 6 months preceding DSA development and during the entire duration of follow up in patients without DSA and found no difference; this was the case for patients in the low and high risk groups (other than for low HLA-DQ risk patients where patients with DSA had higher mean CNI levels) (**Figure S3B**). Finally, the intra-patient variability (IPV) of CNI levels was similar in patients who did and did not develop DSA (data not shown).

**Figure 3:**
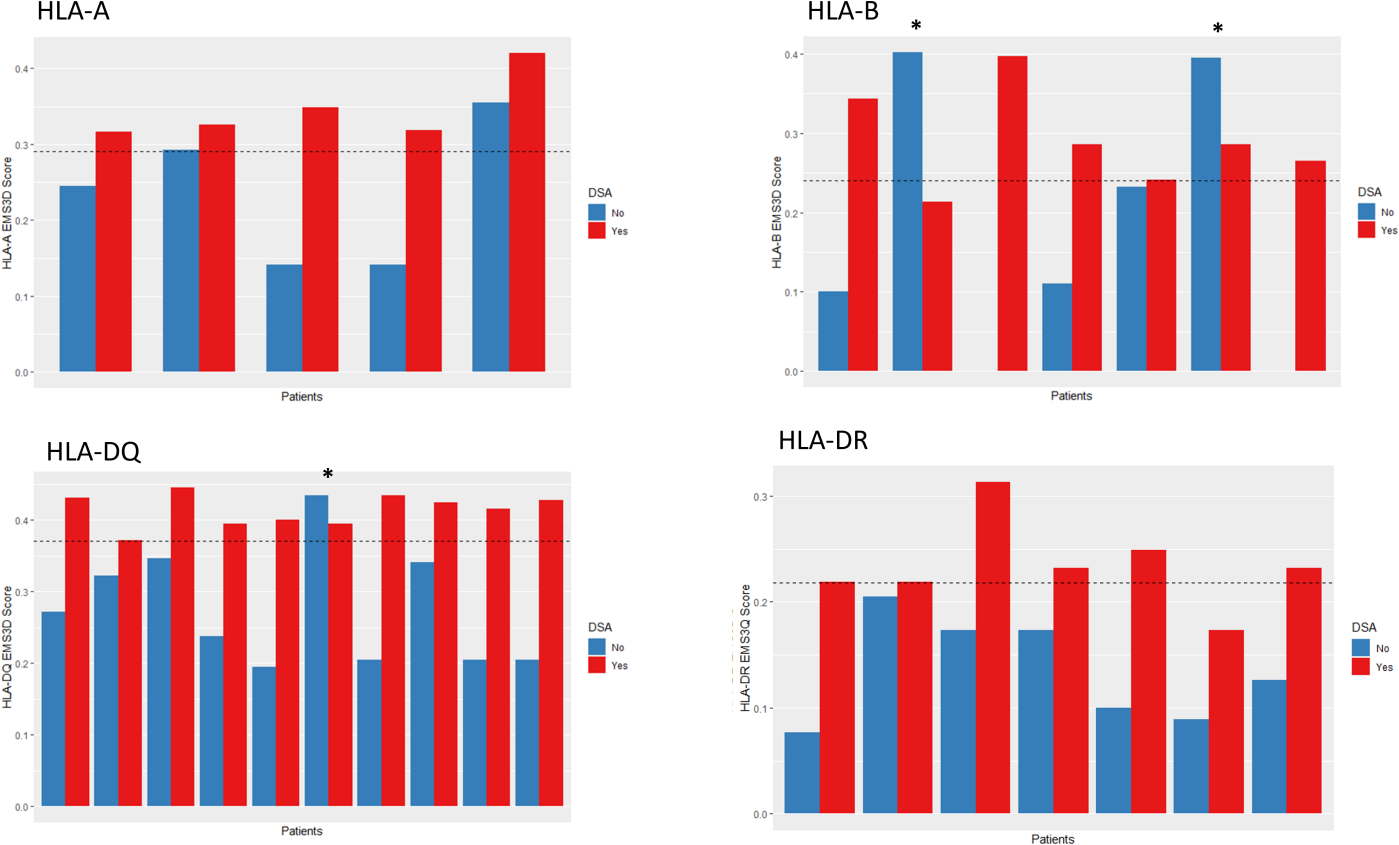
EMS3D scores for each allele in patients with 2MM1DSA (2 antigen mismatch 1 DSA). * denotes DSA which targeted the lower scoring allele. Some alleles had scores of zero. The dotted line represents the molecular risk threshold based on the Youden index from the single molecule analysis.

Notably, in patients with two allelic mismatches within a locus, the DSA targeted the allele with the highest molMM score in the majority of cases (HLA-A: 5/5, HLA-B: 5/7, HLA-DQ: 9/10, HLA-DR: 7/7, denominator denotes the number of patients with two allelic HLA mismatches in the respective locus and one DSA; results were the same for all molMM methods, **Figure 3** depicts molMM scores and DSA target using the EMS3D score). In two out of the total of three mis-classified cases the DSA targeted HLA with molMM score above the Youden index.

### T-cell mediated rejection and antibody-mediated rejection

For TCMR, we focused on late acute rejection, defined as occurring >6 months post-transplant, due to its importance in allograft failure (28). Overall, the incidence of late TCMR (23 patients, 13%) and of ABMR (11 patients, 6%), as diagnosed on ‘for-cause’ biopsies, in this cohort was low. Notwithstanding this limitation (especially for ABMR models), we used multivariable Cox models to investigate the relationship between donor-recipient HLA incompatibility and rejection risk (**Table 2**). Neither the number of antMM nor any of the molMM scores for individual HLA loci, HLA class I or HLA class II were associated with TCMR risk in this cohort.

**Table 2:** Survival analysis for biopsy-proven rejection outcomes: A) Late TCMR (>6 months post-transplant, events N = 23, at risk N = 177); B) ABMR (events N = 11, at risk N = 177).

A) Late TCMR (>6 months), events N = 23					
	Antigen	AAMS	EMS3D	netMHC	netMHC1k
Class I (sum)	0.83 (0.54-1.27)	0.96 (0.92-1.01)	0.79 (0.31-1.98)	0.96 (0.89-1.03)	0.98 (0.96-1.00)
HLA-A	0.76 (0.39-1.50)	0.94 (0.87-1.01)	0.20 (0.03-1.51)	0.92 (0.82-1.04)	0.97 (0.95-1.01)
HLA-B	0.81 (0.40-1.65)	1.01 (0.91-1.13)	1.74 (0.16-18.4)	1.00 (0.88-1.14)	0.97 (0.94-1.01)
Class II (sum)	1.50 (0.92-2.44)	1.01 (1.00-1.02)	1.07 (0.32-3.62)	1.01 (0.96-1.06)	1.00 (0.99-1.01)
HLA-DQ	1.81 (0.93-3.51)	1.02 (1.00-1.04)	3.06 (0.42-22.4)	1.04 (0.99-1.11)	1.01 (1.00-1.02)
HLA-DR	1.22 (0.56-2.63)	1.00 (0.97-1.03)	0.41 (0.053.14)	0.85 (0.71-1.03)	0.99 (0.98-1.01)
B) ABMR, events N = 11					
	Antigen	AAMS	EMS3D	netMHC	netMHC1k
Class I (sum)	1.28 (0.58-2.86)	0.97 (0.91-1.04)	0.66 (0.13-3.36)	1.01 (0.91-1.12)	0.99 (0.96-1.02)
HLA-A	1.78 (0.60-5.31)	1.01 (0.93-1.11)	0.81 (0.05-12.8)	1.01 (0.88-1.16)	0.99 (0.95-1.03)
HLA-B	0.87 (0.26-2.86)	0.95 (0.78-1.15)	2.08 (0.03-130)	1.00 (0.80-1.24)	0.98 (0.93-1.04)
Class II (sum)	1.32 (0.61-2.87)	1.02 (1.00-1.04)*	7.5 (1.01-55.7)*	1.06 (0.99-1.12)	1.01 (1.00-1.02)
HLA-DQ	1.15 (0.42-3.16)	1.02 (1.00-1.05)*	25 (1.01-640)*	1.08 (1.00-1.16)*	1.01 (1.00-1.03)
HLA-DR	1.64 (0.48-5.60)	1.03 (0.99-1.07)	7.22 (0.25-206)	0.99 (0.78-1.25)	1.01 (0.99-1.04)

Individual HLA molecular mismatch scores are summed together for class I, class II, HLA-DQ and HLA-DR. Each line represents a separate model adjusted for recipient age, recipient ethnicity, graft number, cold ischaemia time and percentage panel reactive antibodies. *p < 0.05.

In ABMR models, only HLA-DQ mismatching, assessed using the total molMM scores for mismatches in this locus, was significantly associated with ABMR-free survival for AAMS (HR: 1.02, 95% CI: 1.00-1.05 per amino acid mismatch), EMS3D (HR: 25, 95% CI: 1.01-640 per unit increase), and netMHC (HR: 1.08, 95% CI: 1.00-1.16 per donor peptide) (**Table 2**). However, fitting multivariable models using HLA-DQ low versus high EMS3D risk group molMM stratification did not show a significant association with ABMR (adjusted HR: 2.3, 95% CI: 0.58-9.6 for EMS3D; **Figure S4**). The same was the case after HLA-DQ risk stratification using the other molMM methods (data not shown). Nevertheless, the small number of ABMR cases in this cohort did not enable a robust assessment of risk stratification at the patient level.

Taken together, the above analyses indicate that molecular HLA mismatching was superior to serological HLA mismatching at predicting the risk of DSA development and of ABMR. No single molMM clearly outperformed the others, although a robust comparison of molecular mismatch methods was not possible in this relatively small patient cohort. Overall, EMS3D was the most consistent method for stratifying risk of primary humoral alloimmunity in this patient cohort.

### Graft function deterioration

**Figure 4:**
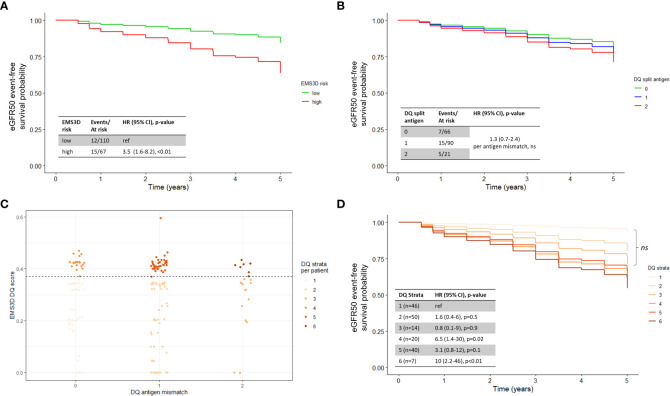
Survival analysis for eGFR50 outcome, adjusted for baseline eGFR, recipient age, donor age and transplant number, as per **Table 3**. Adjusted survival curves were visualised using average weighting in the survminer R package (version 0.4.9) (29). **(A)** EMS3D HLA-DQ risk categories; **(B)** Serological HLA mismatching. **(C)** EMS3D values for each HLA-DQAB antigen mismatch. Each dot represents one patient and the maximum EMS3D score is presented. The EMS3D result of 0.37 was used as the threshold to define patients into high/low molecular risk, based on the Youden-index from DSA ROC analysis. Patients were therefore sub-divided into DQ strata as follows: 1 – 0MM/low, 2 – 1MM/low, 3 – 2MM/low, 4 – 0MM/high, 5 – 1MM/high, 6 – 2MM/high. **(D)** Survival analysis for eGFR50 outcome based on HLA-DQ strata. *ns* – not significant.

During the study timeframe, only five patients lost their grafts – two were associated with ABMR, one each due to BK nephropathy and recurrence of primary disease, and one not stated. As a surrogate outcome for long-term graft loss, we used eGFR50 defined as persistent 50% reduction in graft function from the baseline eGFR at month 3. This outcome was met in 27 (15%) patients. Based on the above results, we stratified patients using the EMS3D risk threshold from the DSA analysis in a ‘time-zero’ Cox model (i.e. utilising information available at the time of transplantation) adjusted for donor and recipient factors (**Table 3**). As expected, donor and recipient age, baseline eGFR and re-transplantation were significant predictors of eGFR50 (**Table S4**). Patients with high risk HLA-DQ mismatches, as assessed using EMS3D, were more likely to reach the eGFR50 endpoint (high versus low risk: adjHR 3.5, 95% CI 1.6-8.2, p<0.005) (**Figure 4A**), whereas molecular mismatching at HLA-A, -B and -DR was not associated with this outcome. Importantly, analysis based on serological HLA mismatches did not show any significant associations with eGFR50-free survival for any HLA-locus (**Figure 4B** shows the adjusted survival curve for HLA-DQ). Of note, there were more patients stratified into the low risk EMS3D group (110 patients) than patients with zero split antigen mismatches (66 patients). Accounting for IPV of CNI levels did not significantly change the results. To assess whether the effect of high risk HLA-DQ mismatching on the eGFR50 outcome is no longer apparent when graft rejection after transplantation is accounted for, we next included rejection episodes as time-varying events in the above ‘time-zero’ multivariable model. As might be expected, TCMR and ABMR were significantly associated with graft function deterioration. Nevertheless, the association of high risk EMS3D HLA-DQ mismatching with eGFR50 remained significant (high versus low risk: adjHR 2.9, 95% CI 1.2-7.0, p<0.05) in this model, suggesting an independent effect beyond rejection episodes detected on ‘for-cause’ graft biopsies (**Table S5**).

**Table 3:** Multivariable Cox proportional hazards survival analysis for eGFR50 outcome, comparing antigen mismatch versus molecular mismatch risk thresholds using EMS3D.

	Events/At risk	Antigen Mismatch (0-2)	EMS3D risk strata (high versus low)
HLA-A	27/177	0.6 (0.3-1.1)	1.3 (0.6-2.9)
HLA-B	27/177	1.0 (0.5-2.0)	0.7 (0.3-1.6)
HLA-DQ	27/177	1.3 (0.7-2.4)	3.5 (1.6-8.2)**
HLA-DR	27/177	1.4 (0.7-2.8)	0.5 (0.2-1.4)

Each line is a separate model, adjusted for baseline eGFR, recipient age, donor age and transplant number. 27 patients met the survival outcome out of 177 at risk. **p < 0.01.

To delineate how molMM might enhance patient risk stratification, we next examined the relationship between traditional antigen HLA-DQ mismatching versus molecular HLA-DQ mismatching and graft function deterioration. Within each level of antigen mismatch, there was a wide range of EMS3D scores (**Figure 4C**). We stratified each antigen mismatch into low or high risk, based on the EMS3D Youden index derived from the DSA analysis above, to generate six strata, i.e. two strata (low- or high-EMS3D) per HLA-DQ antigen mismatch (0–2) (**Figure 4D**). Patients with low risk HLA-DQ mismatches had equivalent eGFR50-free survival regardless of the number of antigen mismatches present (0-MM/low-EMS3D, 1-MM/low-EMS3D and 2-MM/low-EMS3D clustered together, p>0.1), whereas patients with high-risk antigenic mismatches had 3-10 fold higher odds of graft deterioration (using the 0-MM/low-EMS3D group as reference) (**Figure 4D**). Patients with 0-MM/high-EMS3D had worse eGFR50-free survival compared to patients with 2-MM/low-EMS3D. Taken together, the above results indicate that patient risk for graft function deterioration can be stratified more accurately using molecular HLA-DQ mismatching rather than antigenic mismatching.

**Figure 5:**
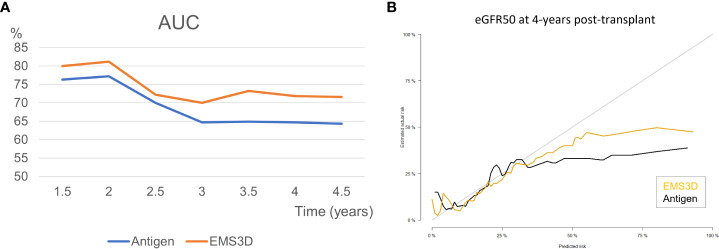
Model discrimination and calibration for eGFR50 outcome using HLA-DQ EMS3D versus HLA-DQ serological mismatching. **(A)** Time-dependent AUC (higher percentage is better); **(B)** calibration curve for risk prediction at 4-years post-transplant. The 45 degree line represents matched predicted and actual risk. Models were cross-validated internally using leave-one-out validation with 200 bootstrap iterations (30).

We further compared the performance indices of risk stratification models for the eGFR50 outcome using EMS3D (high and low risk) versus antMM. Model discrimination (the performance of the multivariable ‘time-zero’ model in correctly ranking patients according to risk of eGFR50 outcome) was assessed using time-dependent AUC. Stratification with EMS3D had consistently higher AUCs throughout the study period (81% at 2 years, and 70-73% after 2 years post-transplant) compared to antMM (77% at 2 years, reducing to 64-65% after 3 years post-transplant) (**Figure 5A**). **Figure 5B** shows the model calibration plots, derived using leave-one-out cross-validation, for prediction of the eGFR50 outcome at 4-years post-transplant. The 45-degree line represents a match between the predicted risk and actual risk. Overall, both models had similar calibrated risk prediction for patients at low risk but the EMS3D model had better calibration performance for patients at higher risk of graft function deterioration.

## Discussion

In this study, we compared four methods of molecular HLA mismatch versus traditional HLA antigenic mismatch in assessing risk of alloimmune outcomes and graft function deterioration after paediatric kidney transplantation. We show that at the individual HLA level, DSAs targeted the highest scoring molMM alleles, whereas at the patient level, molMM scores were better predictors of *de novo* DSA against donor HLA-A, -B, -DR, and -DQ than serological HLA mismatching. In this relatively small cohort, no single molMM clearly outperformed the others, although EMS3D was the most consistent predictor of DSA development across all HLA loci examined. At the population level, HLA-DQ mismatching assessed using molMM for risk stratification was associated with long-term graft function deterioration (eGFR50) providing good model discrimination, whereas traditional antMM was a poor predictor of this outcome. Taken together, this study provides promising evidence for the clinical utility of molecular HLA mismatching to assess donor HLA immunogenicity, risk for primary humoral alloimmunity and adverse outcomes in the paediatric kidney transplantation setting.

Our main objective was to assess the utility of molecular HLA mismatching for risk stratification rather than just establish its association with humoral alloimmunity. We used the previously published ‘single molecule method’ to define molMM risk thresholds and risk stratify patients (10). This contrasts with other studies which used the sum of molecular HLA mismatch scores within or across HLA loci to investigate associations between molMM and alloimmune outcomes (12, 13, 26). In support of the immunological basis of the ‘single molecule method’, we showed that in patients with two allelic mismatches within a locus, the alloantibody response targeted the HLA with the higher molecular score in the majority of cases. In cases where the DSA targeted the lower scoring HLA mismatch, the relevant molMM score was still above the established risk threshold in all but one case (HLA-B). This concept of 2MM1DSA to study HLA mismatch immunogenicity was introduced by Tambur et al. who also found that HLA-DQ DSA preferentially targeted the highest scoring allele as assessed using AAMS (26/29 patients) (17). Of note, we did not observe patients with DSAs against both alleles (2MM2DSA) in our cohort. Multiple studies have also shown that DSA can target HLA mismatches with low molMM scores, albeit this is relatively uncommon and the relative immunogenicity of low risk HLA mismatches might be easier to control with immunosuppression (31). Nevertheless, more research is required before using molecular HLA mismatch strategies to design immunosuppression minimisation regimens (13, 17).

Our study compared AAMS, EMS3D and netMHCIIpan algorithms for molecular HLA mismatch assessment using current up-to-date algorithms. Amino acid polymorphisms within the HLA extra-cellular domain were assessed in all methods. We elected to not formally evaluate HLAMatchmaker as we have previously shown that eplet load correlates highly with AAMS (R^2 =^ 0.95 and 0.96 for HLA-DQ and DR, respectively) (11). Eplet scores were utilised in other paediatric studies (encompassing 59-196 patients) which reported associations with Class I and Class II DSA, although association with graft function was not examined (32–34). For molecular mismatch assessment using netMHCIIpan, recipient HLA-DQ, DRB1 and DRB3/4/5 molecules were used to examine donor peptide presentation. NetMHC1k did not have a better performance compared to netMHC at predicting *de novo* DSA and it was notable that netMHC risk stratification was superior to netMHC1k for HLA-DR DSA. This finding suggests that the more stringent criteria used in the formal implementation of the netMHCIIpan v4.0 algorithm for identification of potential CD4^+^ T-cell epitopes is potentially valuable and should be further evaluated in future studies (24). Nevertheless, a more relevant assessment of this algorithm would have involved prediction of alloreactive T-cell priming post-transplantation, which was not possible in this study. In this regard, a study by Meneghini et al. suggested that molecular HLA incompatibility, as assessed using the global PIRCHE-II score (summation of scores for all donor HLA), may predict *de novo* donor-specific T-cell priming after kidney transplantation, especially when less stringent peptide affinity thresholds are used (35). However, and in contrast to previous studies, we could not demonstrate an association between molecular (or antigenic) HLA mismatch and TCMR in this study, neither using the netMHCIIpan approach nor the AAMS and EMS3D algorithms (10, 13). As was the case in previous studies, netMHC1k correlated with amino acid mismatching (R^2 =^ 0.67-0.87, allelic matches excluded) and, therefore, the association of netMHC1k with DSA development may reflect in part that this score provides an assessment of donor-recipient HLA dissimilarity at the sequence level rather than a true assessment of T-cell alloreactivity (26). Molecular HLA mismatch model discrimination for class II DSA development was similar among all algorithms. Notably, the correlation between molMM scores was highest for HLA class II mismatches (0.71-0.80 for HLA-DR and 0.71-0.87 for HLA-DQ). Model discrimination was similar among molMM methods for HLA-A DSA prediction, but EMS3D was the best predictor of DSA development against HLA-B mismatches. Importantly, EMS3D scores correlated poorly with AAMS and netMHC1k scores for HLA class I loci, and EMS3D provided more meaningful patient stratification for risk of *de novo* HLA-A, -B, -DR, and DQ DSA with significant differences in DSA-free survival between low and high risk groups, compared to AAMS and netMHCIIpan algorithms. EMS3D model discrimination for *de novo* DSA prediction was consistent across all HLA loci with AUCs above 0.7, which is comparable to results reported in the literature, considering we excluded allelic HLA matches in our analyses (10, 11). Nevertheless, it should be acknowledged that the relatively small size of this study did not enable a robust comparison of molecular HLA mismatch methods.

This study identified an association between HLA-DQ molecular mismatching (as assessed by AAMS, EMS3D or netMHC algorithms) and ABMR, but the low incidence of rejection episodes did not enable investigation of the capacity of molMM algorithms for risk stratification at the patient level. Additionally, rejection was only confirmed with for-cause biopsies and subclinical rejection could have been missed (36). Importantly though, we showed that HLA-DQ molecular mismatching was associated with long-term graft function deterioration (eGFR50), after adjustment for patient and donor factors that are available at the time of transplantation. This was also the case after adjusting for post-transplant TCMR and ABMR episodes, suggesting an independent effect of high risk HLA-DQ mismatching on long-term function; it is tempting to speculate that this effect may be mediated through subclinical rejection. As proof of principle, we used EMS3D to effectively stratify patients into high/low risk groups. To our knowledge, our study is the first to demonstrate superiority of the molMM approach over traditional antMM for the prediction of this outcome. Patients clustered according to the molMM risk category regardless of the underlying antigenic HLA differences. There were 110 patients in the low EMS3D-risk group compared to 66 patients in the zero antigen mismatch group, which suggests that molecular mismatching might identify more potential donor-recipient combinations at low risk of adverse graft outcomes. This would be an important result to validate in larger studies, because it may pave the way towards incorporation of HLA-DQ mismatching (high or low EMS3D molecular risk) in organ allocation algorithms, provided equity of access and waiting times are not jeopardised (37).

This study aligns with the STAR (Sensitisation in transplantation: Assessment of Risk) Working Group research priority of assessing molMM for primary alloimmune responses (38, 39). In particular, paediatric recipients represent the ideal study group due to low levels of prior sensitisation and stand to benefit the most due to their lifetime needs for more than one transplant. Large paediatric registry studies have shown that graft survival is incrementally worse with increasing number of HLA antigenic mismatches (1, 40–42). In smaller studies, the effect of HLA mismatching is less evident (43, 44). HLA mismatching based on traditional antigen enumeration does not account for the underlying genetic heterogeneity of the HLA molecule, and therefore larger studies are required to show aggregate differences in patient populations. In this relatively small study, we were already able to show an important association of HLA-DQ mismatching, as assessed using molMM, with long-term graft function. In adults, the effect of HLA-DQ mismatching on graft survival using antMM is nuanced. A significant association with HLA-DQ antigen mismatching has only been identified when analysing subpopulations, i.e. living donors and deceased donors with short cold ischaemic times (45, 46). There were also statistically significant interactions between HLA-DR and DQ (45, 46). We could not identify an association between HLA-DR mismatching and eGFR50 in our study (47). Our study therefore adds to a growing body of evidence for incorporating HLA-DQ, specifically using molMM, into assessments of patient alloimmune risk and of long-term graft outcomes (10, 13, 37, 44, 48).

It is important to recognise the limitations of this study. The number of included patients was relatively small commensurate with the low number of paediatric kidney transplants performed in Europe, compared with adult kidney transplantation, which poses difficulties for studies of this kind. To increase the number of patients, we utilised the CERTAIN registry which is an international collaborative (CERTAIN data is vetted before being accepted). All patients with data on prospective HLA antibody testing were included and no prior sample size calculation was performed, but we accept that there is a risk of bias relating to patient inclusion. We acknowledge that DSA testing was performed using different protocols for serum processing and MFI thresholds, although this reflects current clinical reality. Additionally, the number of patients with biopsy-proven rejection (TCMR and ABMR) were low, and, therefore, the study was underpowered in this regard. Immunosuppression protocols and management of patients who developed DSA were dependent on local practices and is a potential source of bias in this study. HLA imputation was required to obtain the two-field (high-resolution) HLA typing, although this has been shown to be more accurate in the Caucasian population which represented the majority of donors and recipients in our study (49, 50). We acknowledge that further studies are required to externally validate the molecular HLA mismatch cut offs identified in our study and in this regard, our study was not intended to be definitive. Our population was a relatively well-matched Caucasian population with low levels of HLA sensitisation and applicability of our findings in highly sensitised patients requires further studies (17). It is notable that various thresholds have been described in the literature and an absolute cut-off applicable to all populations might be unlikely (10, 17). The analysis workflow described herein allows the risk thresholds to be calculated and applied for local population HLA allelic frequencies. We used eGFR50 as a surrogate marker of long-term graft loss, and future studies will need to validate the applicability of this surrogate endpoint in paediatric kidney transplantation (51). Finally, previous studies have shown the importance of immunosuppression levels and non-adherence in modulating the risk from molecular HLA mismatch; we have addressed these potential confounders within the confines of our dataset, although more detailed assessment of immunosuppression and of relevant treatment protocols would be advantageous (15, 31).

In summary, this study supports molecular HLA mismatching as a significant advance over current serological HLA mismatching for the prediction of humoral alloimmunity and identifies molecular HLA-DQ mismatching as a potential prognostic biomarker of long-term graft dysfunction in paediatric kidney transplantation.

## Methods

### Data source

Patients were recruited from the web-based CERTAIN Registry, which is an international research registry with funding for patient entry (52). To facilitate recruitment, data is exchanged bi-directionally with Eurotransplant and ERA-EDTA Registry for centres within Europe. The registry collects clinical and laboratory data of paediatric kidney transplant recipients in a detailed and comprehensive manner every 3 months in the first year and six-monthly thereafter. This allows a thorough patient characterization in general and also of specific patient subcohorts (52). Written informed consent was obtained from all parents/guardians to participate in the registry, with assent from patients when appropriate for their age. The CERTAIN Registry has been approved by the respective ethics committee of each contributing centre and is kept in full accordance with the principles of the Declaration of Helsinki and Good Clinical Practice guidelines. The study was designed, analysed and reported according to the STROBE guidelines (https://www.strobe-statement.org).

### Patient population

We included patients from centres which routinely performed prospective monitoring of post-transplant HLA antibodies, at minimum within the first year post-transplant and yearly thereafter. No exclusion criteria were applied. HLA antibody testing was performed following local protocols, using screening panels followed by single antigen HLA specific beads. HLA antibody positivity was defined using local thresholds (**Table S6**). Only *de novo* donor-specific HLA antibodies (DSA) were included in this study, and HLA specificity was assigned at the allelic level by comparing donor and recipient HLA allelic types and referencing results from individual allelic beads within single antigen panels. Histology reports were verified by an independent histopathologist based on the Banff 2017 criteria (53). The CERTAIN Registry was accessed at 15/05/2020. The data were not sufficient to perform DSA analysis for HLA-C, but otherwise had no missing data.

### Molecular HLA algorithms

HLA typing in the registry was provided for HLA-A, -B, -DR and -DQ at the split serotype level along with donor/recipient ethnicity. Allelic imputation was performed using population specific haplotype frequency data from the National Marrow Donor Program for HLA-A, -B, -C, -DQA1, -DQB1, -DRB1 and DRB3/4/5 loci (54, 55). The probable haplotype pair was assigned for each donor/recipient genotypes.

Amino Acid Mismatch Score (AAMS) and Electrostatic Mismatch Score 3D (EMS3D) calculations: The number of amino acid mismatches present on donor HLA was calculated using the Cambridge HLA Immunogenicity algorithm, as previously described (7, 8). Similarly, electrostatic potential differences on the surface of donor compared to recipient HLA were calculated using the EMS3D algorithm, as previously described (7, 8, 20–22, 56).

NetMHCIIpan (version 4.0) was used to predict the affinity and percentage rank of all potential 15-mer peptides present in donor HLA extracellular sequences (which were absent from recipient sequences), using recipient HLA-DRB1, HLA-DRB3/4/5 and HLA-DQ as presenting molecules. The resultant 9-mer core, affinity and percentage rank was used to enumerate the number of unique 9-mer cores from all potential peptides per donor HLA that either met a 1000 nM threshold (abbreviated netMHC1k) or both of a 500 nM threshold and were in the top 2% percentage rank (abbreviated netMHC). The former (1000 nM) peptide threshold is that employed by the PIRCHE algorithm (6, 24).

### Statistical analysis

We analysed the ability of the molecular HLA mismatch scores to predict *de novo* locus-specific DSA development using the ‘single molecule’ method (10). Briefly, each donor HLA mismatch was scored and correlated to DSA development against that molecule specifically, independent of patient specific effects. Thresholds were derived from the Youden index using receiver operating characteristic (ROC) curve analysis for each HLA locus and high/low molMM categories were defined.

We then applied the molecular mismatch scores to predict DSA development at the patient level. In patients with two HLA mismatches within a locus, the highest scoring mismatch was used to represent the patient’s molMM score. We assessed the ability of molMM scores to predict locus-specific DSA development at the patient level and used the area under ROC curve (AUC) to compare the discrimination ability of each molMM score. Pair-wise comparisons between models were performed using the “DeLong” method in pROC package (version 1.18.0) in R (57).

We used cumulative event curves and Cox proportional hazards models for time to event analyses. Patients were right-censored at time of last follow-up or patient death. We performed univariate analysis for each locus-specific DSA outcome using the molecular risk thresholds and compared groups using log-rank tests. For TCMR and ABMR, we performed multi-variate analysis with molMM scores as the main predictor and adjusted for recipient age, recipient ethnicity, graft number, cold ischaemic time and panel reactive antibodies (%), defined *a priori*. Patient adherence was assessed using the surrogate marker, intra-patient variation (IPV) in immunosuppression levels (28). All patients were prescribed tacrolimus except four patients using ciclosporin. CNI levels were taken from month 6 post-transplant and were available every 6 months. Due to the limited number of measurements, CNI levels for the whole duration of follow-up was used to calculate the IPV using the mean absolute deviation. A threshold of 25% was used to categorise patients into high/low IPV as previously published (28). We also analysed CNI levels in the months preceding DSA formation. For the four patients on ciclosporin, the level was divided by 10 to approximate tacrolimus levels. We compared CNI levels between positive and negative patients for each molMM risk group (high/low) using the Mann Whitney test. We also assessed the number of times the CNI level was above 5ng/ml for patients with and without DSA, as described by Wiebe et al. (31).

Graft function deterioration was modelled using the eGFR50 outcome, defined as persistent 50% reduction in graft function (on two consecutive results six months apart) from the baseline eGFR taken at month 3. As the aim of the study was to assess the predictive ability of HLA mismatching, we first constructed a base model using donor and recipient characteristics (**Table S4**). Four variables were kept in the step-wise multiple regression analysis: baseline eGFR, recipient age, donor age and transplant number, which were used as covariates for further analyses of eGFR50 outcome. Statistical analyses were performed using ‘survival’ package (version 3.2-13) in R (version 4.1.2) (58, 59) Model accuracy and discrimination were analysed using time-dependent AUC and calibration plots. Results were validated using leave-one-out internal cross-validation with 200 bootstrap iterations (30) P-values of <0.05 were considered statistically significant and results were considered exploratory as multiple models were fitted.

## Data availability statement

The datasets presented in this article are not readily available. Access to CERTAIN registry data is available by applying through the Scientific Research Committee following Registry Statutes. Requests to access the datasets should be directed to https://certain-registry.kikli.uni-heidelberg.de/.

## Ethics statement

Participation in the CERTAIN Registry is approved by the ethics committee in each center. Written informed consent to participate in the registry was provided by the participants’ legal guardian/next of kin.

## Author contributions

JKi, AF, HC, BT and VK contributed to conception and design of the study. AF, CS, LS, JO, LP, LW, MW, JKo and BT recruited patients and contributed data. AF and KK organized the database. JKi, HC and LG performed the computational and statistical analysis. JKi and VK wrote the first draft of the manuscript. JKi, HC, LG, BT and VK wrote sections of the manuscript. All authors contributed to the article and approved the submitted version.
