# Corrigendum: Molecular HLA mismatching for prediction of primary humoral alloimmunity and graft function deterioration in paediatric kidney transplantation

**DOI:** 10.3389/fimmu.2023.1188527

**Published:** 2023-03-24

**Authors:** Jon Jin Kim, Alexander Fichtner, Hannah C. Copley, Loren Gragert, Caner Süsal, Luca Dello Strologo, Jun Oh, Lars Pape, Lutz T. Weber, Marcus Weitz, Jens König, Kai Krupka, Burkhard Tönshoff, Vasilis Kosmoliaptsis

**Affiliations:** ^1^ Department of Surgery, University of Cambridge, Cambridge, United Kingdom; ^2^ Department of Paediatric Nephrology, Nottingham University Hospital, Nottingham, United Kingdom; ^3^ Department of Pediatrics I, University Children’s Hospital Heidelberg, Heidelberg, Germany; ^4^ School of Medicine, Tulane University, New Orleans, LA, United States; ^5^ Transplantation Immunology, Institute of Immunology, University Hospital Heidelberg, Heidelberg, Germany; ^6^ Renal Transplant Unit Bambino Gesù, Children’s Hospital, Rome, Italy; ^7^ University Hospital Hamburg, Pediatric Nephrology, Hamburg, Germany; ^8^ Clinic for Paediatrics III, Essen University Hospital, Essen, Germany; ^9^ Pediatric Nephrology, Children’s and Adolescents’ Hospital, University Hospital Cologne, Cologne, Germany; ^10^ University Hospital Tübingen, Pediatric Nephrology, Tübingen, Germany; ^11^ Department of General Pediatrics, University Children’s Hospital, Münster, Germany; ^12^ NIHR Blood and Transplant Research Unit in Organ Donation and Transplantation at the University of Cambridge and the NIHR Cambridge Biomedical Research Centre, Cambridge, United Kingdom

**Keywords:** kidney transplantation, HLA mismatch, molecular mismatch, eplets, predictive modeling, antibody mediated rejection (ABMR), donor specific HLA antibodies

In the published article, there was an error in the Funding statement. We missed out an extra funder. The correct Funding statement appears below:

The CERTAIN registry received funding from Astellas and Novartis. The funder was not involved in the study design, collection, analysis, interpretation of data, the writing of this article or the decision to submit it for publication. All authors declare no other competing interests. The authors gratefully acknowledge the funding of the CERTAIN Registry by a grant from the Dietmar Hopp Stiftung, the European Society for Paediatric Nephrology (ESPN) and The German Society for Paediatric Nephrology (GPN). JKi acknowledges funding from a MRC NIHR fellowship (MR/V037900/1). CS acknowledges funding from the European Commision’s Horizon 2020 Research and Innovation Programme (grant no. 952512). VK acknowledges funding from an NIHR Fellowship (PDF-2016-09-065) and as a Paul I. Terasaki Scholar.

VK acknowledges funding from the National Institute for Health Research Blood and Transplant Research Unit (NIHR BTRU, Grant number: NIHR203332) in Organ Donation and Transplantation at the University of Cambridge in collaboration with Newcastle University and in partnership with NHS Blood and Transplant (NHSBT). The views expressed are those of the authors and not necessarily those of the National Health Service, the National Institute for Health Research, the Department of Health or National Health Service Blood and Transplant.

The authors apologize for this error and state that this does not change the scientific conclusions of the article in any way. The original article has been updated.

