# Supplementary material for: Molecular HLA mismatching for prediction of primary humoral alloimmunity and graft function deterioration in paediatric kidney transplantation
Source: Front Immunol. 2023 Mar 15;14:1092335. doi: 10.3389/fimmu.2023.1092335 (PMC10080391; doi:10.3389/fimmu.2023.1092335)
Supplement: Supplementary file 1 [file DataSheet_1.docx]

**Molecular HLA mismatching for prediction of primary humoral alloimmunity and graft function deterioration in paediatric kidney transplantation**

**Supplementary Tables and Figures**

**Table S1:** Details of *de novo* DSA for each patient. Patients 1, 51, and 53 had allele-specific reactivity to DQB1*06:03 bead without reactivity to other DQ6 beads; although the DQB1*06:03 reactivity was noted on repeated measurement of samples obtained at different timepoints, a false positive result cannot be excluded with certainty.
*These patients developed DSA within 3 months post-transplantation. Six of these patients were primary transplants without HLA-specific sensitisation pre-transplantation. The remaining five patients were re-transplants without repeat HLA mismatches to the first graft and with no detectable donor-specific antibodies at the time of transplantation.

**Table S2:** Area under receiver operating characteristic curve results for predicting locus-specific *de novo* DSA at the patient level. Allelic matches were not included in the analysis as no DSA will be formed. For antigen mismatching, the number of locus specific split antigen mismatches was used to predict DSA. The best scores were highlighted in bold. ^a^p<0.05 versus antigen mismatch, ^b^p<0.05 versus netMHC1k

**Table S3:** Youden index derived from receiver operating characteristic area under the curve analyses for the relationship between molecular mismatch scores and *de novo* DSA at each HLA locus. Analysis was performed for each allele using the ‘single molecule’ analysis (10).

**Table S4:** Baseline donor and recipient characteristics associated with eGFR50: variables were assessed using univariate and step-wise, forwards and backwards, multi-variate Cox regression analysis with final variables chosen for best model fit using Akaike Information criterion. Variables were assessed as continuous or categorical. Categorical factors are shown with the first group as the reference. CAKUT: congenital abnormalities of the kidney and urinary tract, *p<0.05 **p<0.01 ***p<0.001

**Table S5:** Combined multivariable Cox proportional hazards survival analysis model for eGFR50 outcome including baseline characteristics, EMS3D HLA-DQ risk stratification and post transplant rejection events: Analysis was performed using Cox regression with rejection (T-cell and antibody mediated) as time-varying covariates, *p<0.05 **p<0.01 ***p<0.001

**Table S6:** Protocol for post-transplant HLA antibody testing for each individual centre

**Figure S1:** ROC curve figures for molecular mismatching at each HLA locus. Analyses were performed using the ‘single molecule’ method as described in the methods section (10).

**Figure S2:** Cumulative event curves for loci-specific DSA for AAMS (A), netMHC (B) and netMHC1k (C) using the same method as shown in Figure 2.

**Figure S3:** Comparison of CNI levels in patients with and without DSA in each molecular risk category (EMS3D): A) Percentage of times CNI were >5 ng/ml prior to DSA formation; B) CNI levels in the 6 months preceding DSA formation. DSA positive (+) versus negative (-) patients were compared using the Mann Whitney test. For DSA- patients, CNI levels were taken from the whole follow-up period. Results are presented as median ± inter-quartile range. Numbers represent number of patients in each category. DSA+ patients within 6 months of transplant were excluded from analysis (A). ns=not significant

**Figure S4:** Cumulative event of ABMR using EMS3D-DQ risk categories. Comparison between groups was performed using log-rank analysis.

| Patient | HLA locus | Antigen MM | Allelic MM | Allelic specificity (serotype) | Time first detected (months post-transplant) |
| --- | --- | --- | --- | --- | --- |
| 1 | DQ | Yes | Yes | DQB1*06:03 (DQ6) | 1* |
| 2 | DQ | Yes | Yes | DQA1*05:05 (DQ7) | 42 |
| 3 | A  DR | Yes | Yes | A*30:01 (A30)  DRB4*01:03 (DR53) | 50  50 |
| 4 | A | Yes | Yes | A*23:01 (A23) | 1* |
| 5 | A  B  DR | Yes | Yes | A*02:01 (A2)  B*44:02 (B44)  DRB1*16:02 (DR16) | 4  4  25 |
| 6 | DQ | Yes | Yes | DQB1*06:02 (DQ6) | 4 |
| 7 | A  DQ | Yes | Yes | A*02:01 (A2)  DQB1*06:04 (DQ6) | 36  36 |
| 8 | DR | Yes | Yes | DRB3*01:01 (DR52) | 25 |
| 9 | B  DR | Yes | Yes | B*57:03 (B57)  DRB5*01:01 (DR51) | 43  43 |
| 10 | DR | Yes | Yes | DRB3*03:01 (DR52) | 1* |
| 11 | A  B  DQ | Yes | Yes | A*23:01 (A23)  B*08:01 (B8)  DQB1*02:01 (DQ2) | 40  40  36 |
| 12 | B | Yes | Yes | B*51:01 (B51) | 50 |
| 13 | B | Yes | Yes | B*15:01 (B62) | 4 |
| 14 | B  DQ  DR | Yes | Yes | B*51:01 (B51)  DQA1*03:02 (DQ7)  DRB4*01:03 (DR53) | 2*  1  2 |
| 15 | DQ | Yes | Yes | DQB1*03:01 (DQ7) | 36 |
| 16 | A | Yes | Yes | A*24:02 (A24) | 1* |
| 17 | DQ | Yes | Yes | DQB1*03:01 (DQ7) | 25 |
| 18 | DQ | Yes | Yes | DQB1*05:03 (DQ5) | 13 |
| 19 | DQ | Yes | Yes | DQB1*06:03 (DQ6) | 1* |
| 20 | DR | Yes | Yes | DRB51*01:02 (DR51) | 45 |
| 21 | A  DR | Yes | Yes | A*68:01 (A68)  DRB4*01:01 (DR53) | 34  48 |
| 22 | A  DQ  DR | Yes | Yes | A*01:01 (A1)  DQA1*01:03 (DQ8)  DRB4*01:01 (DR53) | 61  61  61 |
| 23 | B  DR | Yes | Yes | B*13:02 (B13)  DRB1*01:01 (DR1) | 0.5*  0.5 |
| 24 | A | Yes | Yes | A*26:01 (A26) | 0.5* |
| 25 | A  B  DQ | Yes | Yes | A*25:01 (A25)  B*27:05 (B27)  DQB1*05:01 (DQ5) | 42  42  42 |
| 26 | DR | Yes | Yes | DRB5*01:01 (DR51) | 26 |
| 27 | DQ | Yes | Yes | DQB1*06:03 (DQ6) | 2* |
| 28 | DR | Yes | Yes | DRB1*13:01 (DR13) | 47 |
| 29 | DR | Yes | Yes | DRB4*01:03 (DR53) | 20 |
| 30 | DQ | Yes | Yes | DQB1*02:01 (DQ2) | 7 |
| 31 | DQ | Yes | Yes | DQB1*03:01 (DQ7) | 1* |
| 32 | DQ | Yes | Yes | DQB1*03:01 (DQ7) | 48 |
| 33 | DR | Yes | Yes | DRB1*12:01 (DR12) | 31 |
| 34 | DQ | Yes | Yes | DQA1*05:01 (DQ2) | 16 |
| 35 | B  DR | Yes | Yes | B*44:03 (B44)  DRB1*14:01 (DR14) | 52  52 |
| 36 | B  DQ  DR | Yes | Yes | B*08:01 (B8)  DQB1*03:02 (DQ8)  DRB4*01:03 (DR53) | 38  38  38 |
| 37 | DR | Yes | Yes | DRB5*01:01 (DR51) | 5 |
| 38 | A | Yes | Yes | A*26:01 (A26) | 0.5* |
| 39 | A  B  DQ | Yes | Yes | A*31:01 (A31)  B*08:01 (B8)  DQB1*02:01 (DQ2) | 25  25  22 |
| 40 | A  DQ | Yes | Yes | A*25:01 (A25)  DQA1*05:05 (DQ7) | 37  37 |
| 41 | A  B  DQ  DR | Yes | Yes | A*68:02 (A68)  B*35:01 (B35)  DQB1*02:02 (DQ2)  DRB1*07:01 (DR7) | 8  8  8  8 |
| 42 | DQ | Yes | Yes | DQB1*04:02 (DQ4) | 62 |
| 43 | DQ | Yes | Yes | DQB1*03:01 (DQ7) | 24 |
| 44 | DR | Yes | Yes | DRB4*01:01 (DR53) | 37 |
| 45 | A  B | Yes | Yes | A*02:01 (A2)  B*15:01 (B62) | 17  17 |
| 46 | B  DQ | Yes | Yes | B*08:01 (B8)  DQB1*06:02 (DQ6) | 28  22 |
| 47 | A | Yes | Yes | A*02:01 (A2) | 1* |
| 48 | DQ | Yes | Yes | DQA1*05:05 (DQ7) | 49 |
| 49 | DR | Yes | Yes | DRB1*01:01 (DR1) | 45 |
| 50 | A  B  DR | Yes | Yes | A*02:01 (A2)  B*41:01 (B41)  DRB4*01:01 (DR53) | 9  9  51 |
| 51 | DQ  DR | Yes | Yes | DQB1*06:03 (DQ6)  DRB1*04:03 (DR4) | 2*  2 |
| 52 | A | Yes | Yes | A*11:02 (A11) | 24 |
| 53 | A  DQ | Yes | Yes | A*23:01 (A23)  DQB1*06:03 (DQ6) | 12  12 |
| 54 | DQ | Yes | Yes | DQB1*06:03 (DQ6) | 60 |
| 55 | DQ | Yes | Yes | DQB1*02:01 (DQ2) | 22 |
| 56 | DQ | Yes | Yes | DQB1*06:03 (DQ6) | 37 |

**Table S1:** Details of de novo DSA for each patient. Patients 1, 51, and 53 had allele-specific reactivity to DQB1*06:03 bead without reactivity to other DQ6 beads; although the DQB1*06:03 reactivity was noted on repeated measurement of samples obtained at different timepoints, a false positive result cannot be excluded with certainty.
*These patients developed DSA within 3 months post-transplantation. Six of these patients were primary transplants without HLA-specific sensitisation pre-transplantation. The remaining five patients were re-transplants without repeat HLA mismatches to the first graft and with no detectable donor-specific antibodies at the time of transplantation.

|  | **Antigen** | **AAMS** | **EMS3D** | **netMHC** | **netMHC1k** |
| --- | --- | --- | --- | --- | --- |
| HLA-A (22) | 0.70 | 0.71 | **0.74** | **0.73** | 0.72 |
| HLA-B (15) | 0.52 | 0.67^a^ | **0.72^a,b^** | 0.62 | 0.50 |
| HLA-DQ (30) | 0.52 | 0.72^a^ | **0.75^a^** | 0.66 | **0.77^a^** |
| HLA-DR (22) | 0.58 | **0.76^a^** | 0.71 | **0.76 ^a^** | 0.70 |

**Table S2:** Area under receiver operating characteristic curve results for predicting locus-specific *de novo* DSA at the patient level. Allelic matches were not included in the analysis as no DSA will be formed. For antigen mismatching, the number of locus specific split antigen mismatches was used to predict DSA. The best scores were highlighted in bold. ^a^p<0.05 versus antigen mismatch, ^b^p<0.05 versus netMHC1k

|  | **AAMS** | **EMS3d** | **netMHC** | **netMHC1k** |
| --- | --- | --- | --- | --- |
| **HLA-A** | 9 | 0.293 | 3 | 27 |
| **HLA-B** | 3 | 0.241 | 2 | 19 |
| **HLA-DQ** | 16 | 0.371 | 8 | 41 |
| **HLA-DR** | 8 | 0.219 | 2 | 23 |

**Table S3:** Youden index derived from receiver operating characteristic area under the curve analyses for the relationship between molecular mismatch scores and *de novo* DSA at each HLA locus. Analysis was performed for each allele using the ‘single molecule’ analysis (10).

|  | **Univariate** | **Multivariate** |
| --- | --- | --- |
| **Baseline eGFR (month 3)** | 1.02 (1.01-1.04)*** | 1.02 (1.01-1.04)*** |
| **Recipient age** | 0.91 (0.84-0.97)** | 0.91 (0.83-0.99)* |
| **Recipient gender** (Male/Female) | 1.0 (0.5-2.1) |  |
| **Recipient ethnicity**  (Caucasian/non-Caucasian) | 2.2 (0.5-9.6) |  |
| **Graft number**  (1^st^/2^nd^ or more) | 1.4 (0.6-3.5) | 3.1 (1.1-8.5)* |
| **Pre-emptive** (yes/no) | 0.7 (0.3-1.7) |  |
| **Primary diagnosis:**  CAKUT (ref) Glomerular Other | 0.6 (0.2-1.4)  0.6 (0.2-1.5) |  |
| **Donor Age** | 1.02 (0.99-1.04) | 1.03 (1.00-1.06)* |
| **Cold Ischaemic Time** | 1.0 (1.0-1.0) |  |
| **Panel Reactive Antibody** | 1.0 (1.0-1.0) |  |
| **Intra-patient variability of CNI levels** ≤25% (ref) >25% | 2.6 (1.2-5.7)* | 1.6 (0.68-3.8) |

**Table S4:** Baseline donor and recipient characteristics associated with eGFR50: variables were assessed using univariate and step-wise, forwards and backwards, multi-variate Cox regression analysis with final variables chosen for best model fit using Akaike Information criterion. Variables were assessed as continuous or categorical. Categorical factors are shown with the first group as the reference. CAKUT: congenital abnormalities of the kidney and urinary tract, *p<0.05 **p<0.01 ***p<0.001

|  | **Hazard ratio** |
| --- | --- |
| **EMS3D HLA-DQ risk** (high v low) | 2.9 (1.2-7.0)* |
| **T-cell mediated rejection** | 5.3 (2.1-13.3)*** |
| **Antibody mediated rejection** | 17.4 (4.4-68.6)*** |
| **Baseline eGFR (month 3)** | 1.04 (1.02-1.05)*** |
| **Recipient age** | 0.85 (0.77-0.95)** |
| **Graft number**  (1^st^/2^nd^ or more) | 3.8 (1.3-11.0)* |
| **Donor Age** | 1.04 (1.01-1.07)** |

**Table S5:** Combined multivariable Cox proportional hazards survival analysis model for eGFR50 outcome including baseline characteristics, EMS3D HLA-DQ risk stratification and post transplant rejection events: Analysis was performed using Cox regression with rejection (T-cell and antibody mediated) as time-varying covariates, *p<0.05 **p<0.01 ***p<0.001

| Centre (number of patients) | Post-transplant HLA antibody testing protocol (months) | Serum pre-treatment | MFI cut-off | Vendor |
| --- | --- | --- | --- | --- |
| Heidelberg (85) | 1-3, 6, 12, annually thereafter | EDTA | 500 | OneLambda |
| Hamburg (28) | Annually | Heat inactivation | 1500 | OneLambda |
| Tuebingen (20) | 1-3, 6, 12, annually thereafter | EDTA | 3000 | OneLambda |
| Muenster (19) | 3-6, 12, annually thereafter | No | 1500 | OneLambda |
| Cologne (7) | 6, 12, annually thereafter | EDTA | Individual decision |  |
| Essen (7) | Annually | DTT | 1000 | OneLambda |
| Rome (4) | Annually | No | 1000 | Genprobe |
| Vienna (3) | Annually | No | 1000 | OneLambda |
| Budapest (3) | Annually | No | 500 | OneLambda |
| Berlin (1) | Annually | DTT | 3000 | Genprobe |

**Table S6:** Protocol for post-transplant HLA antibody testing for each individual centre

**
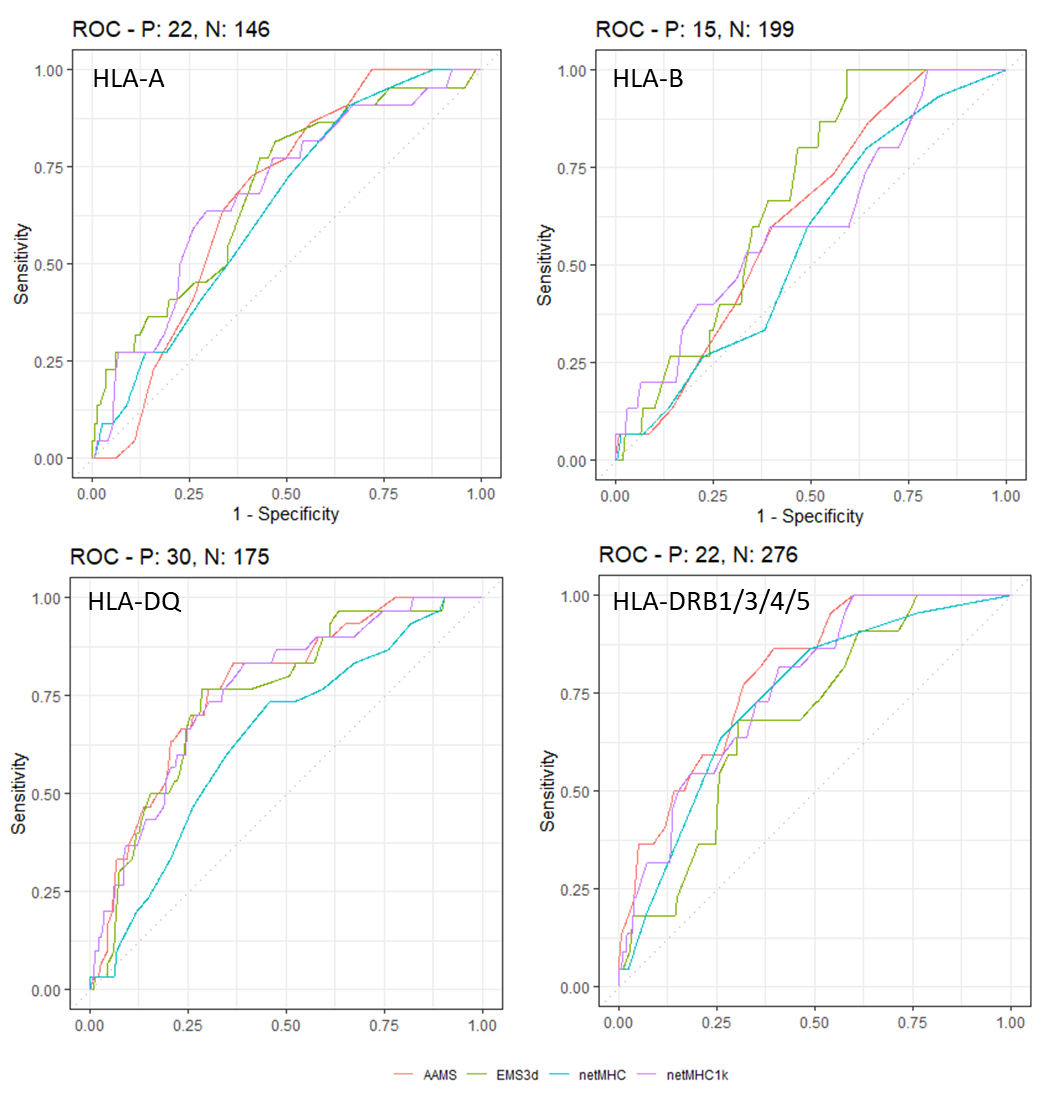
**

**Figure S1:** ROC curve figures for molecular mismatching at each HLA locus. Analyses were performed using the ‘single molecule’ method as described in the methods section.^10^

| **AAMS** | **netMHC** | **netMHC1k** |
| --- | --- | --- |
| HLA-A | | |
| 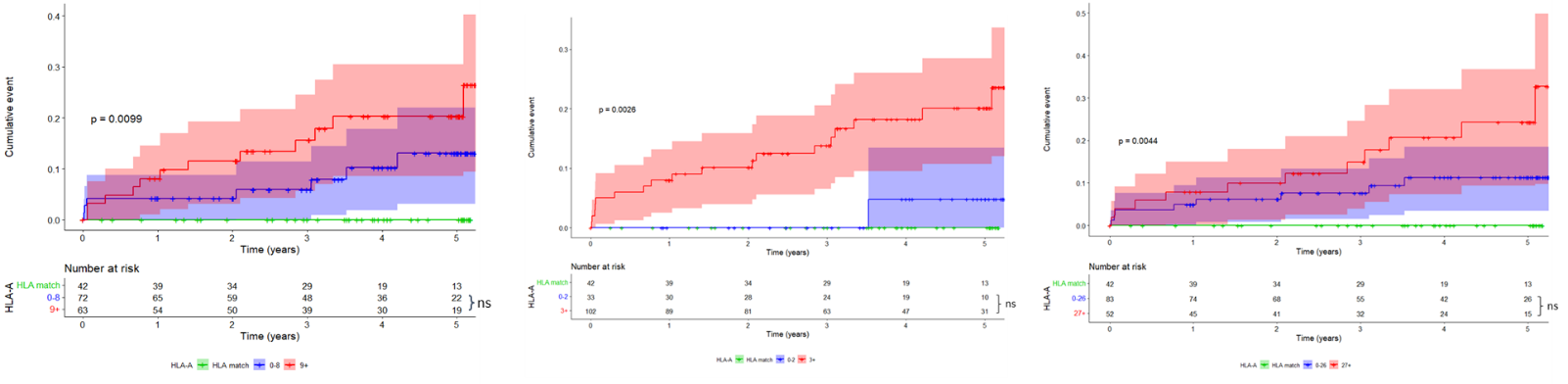 | | |
| HLA-B | | |
| 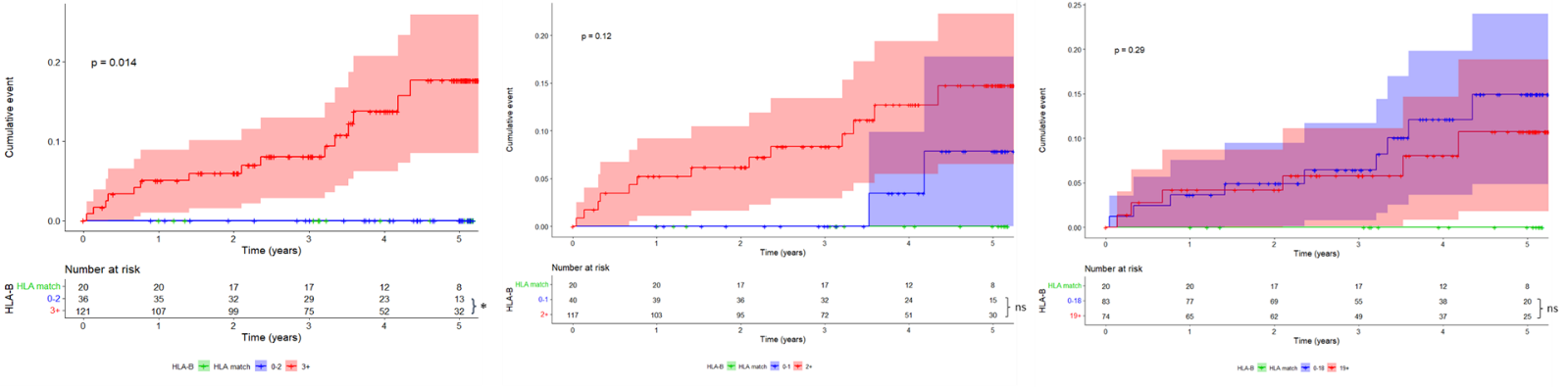 | | |
| HLA-DQ | | |
| 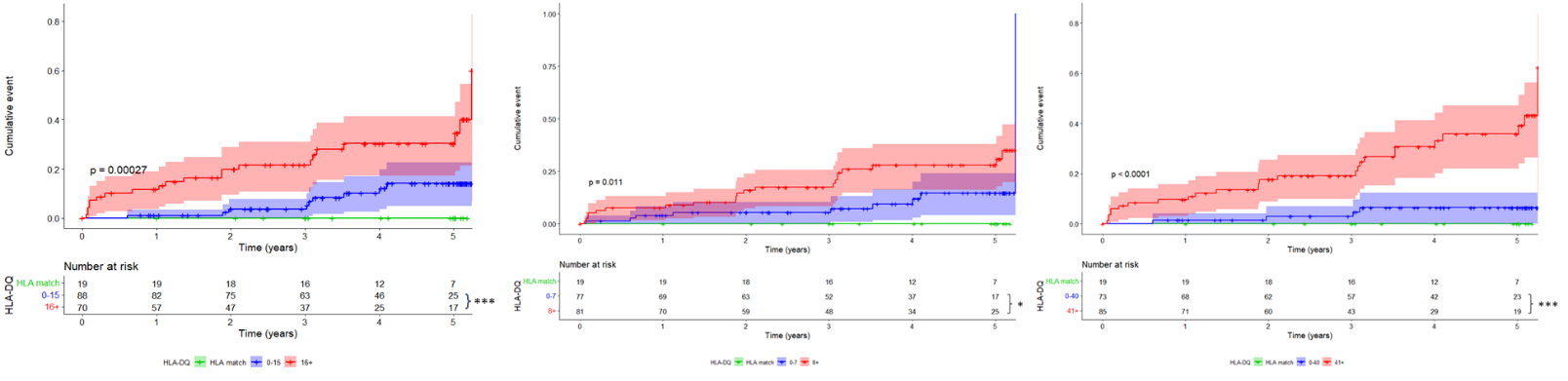 | | |
| HLA-DR | | |
| 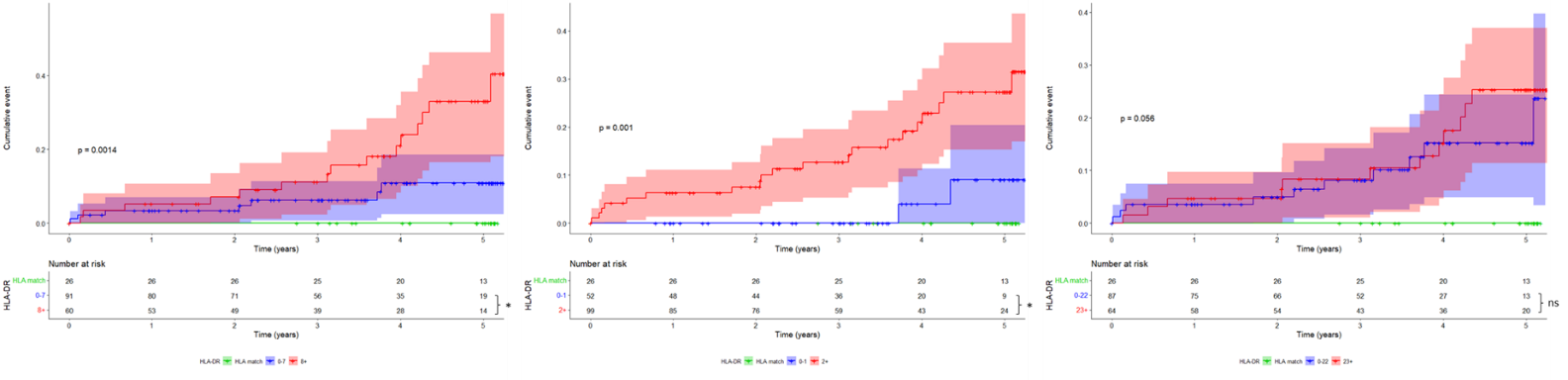 | | |

**Figure S2:** Cumulative event curves for loci-specific DSA for AAMS (A), netMHC (B) and netMHC1k (C) using the same method as shown in Figure 2.

A)


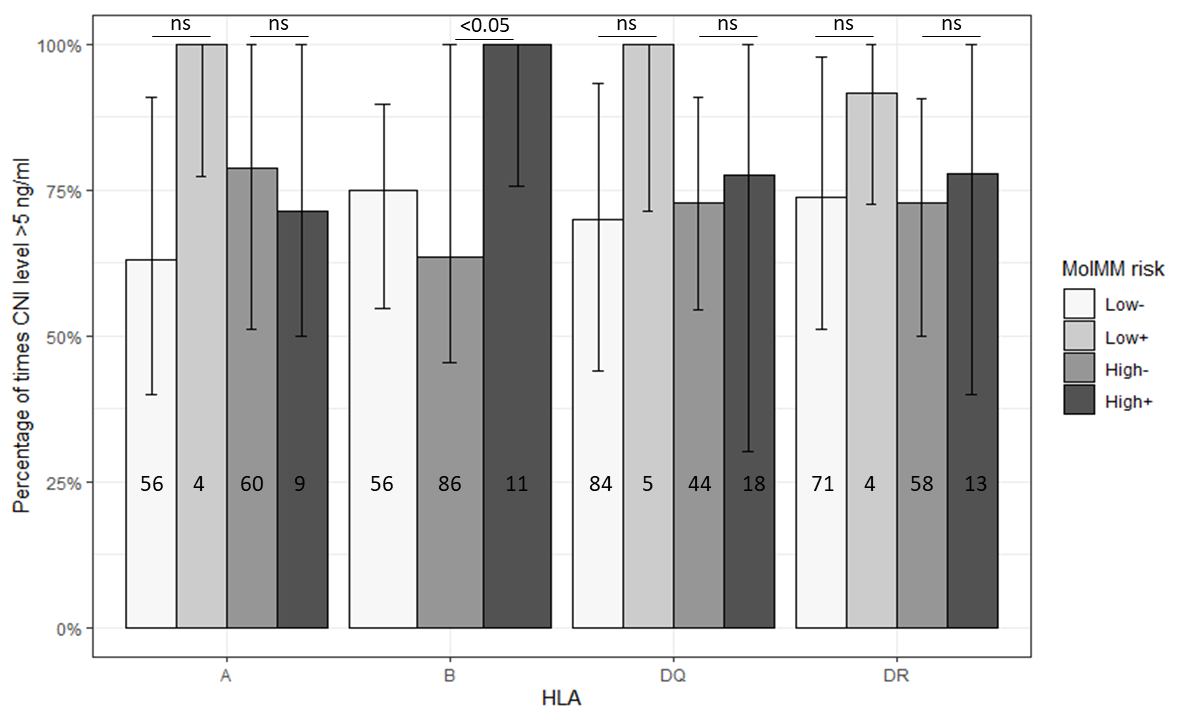


B)


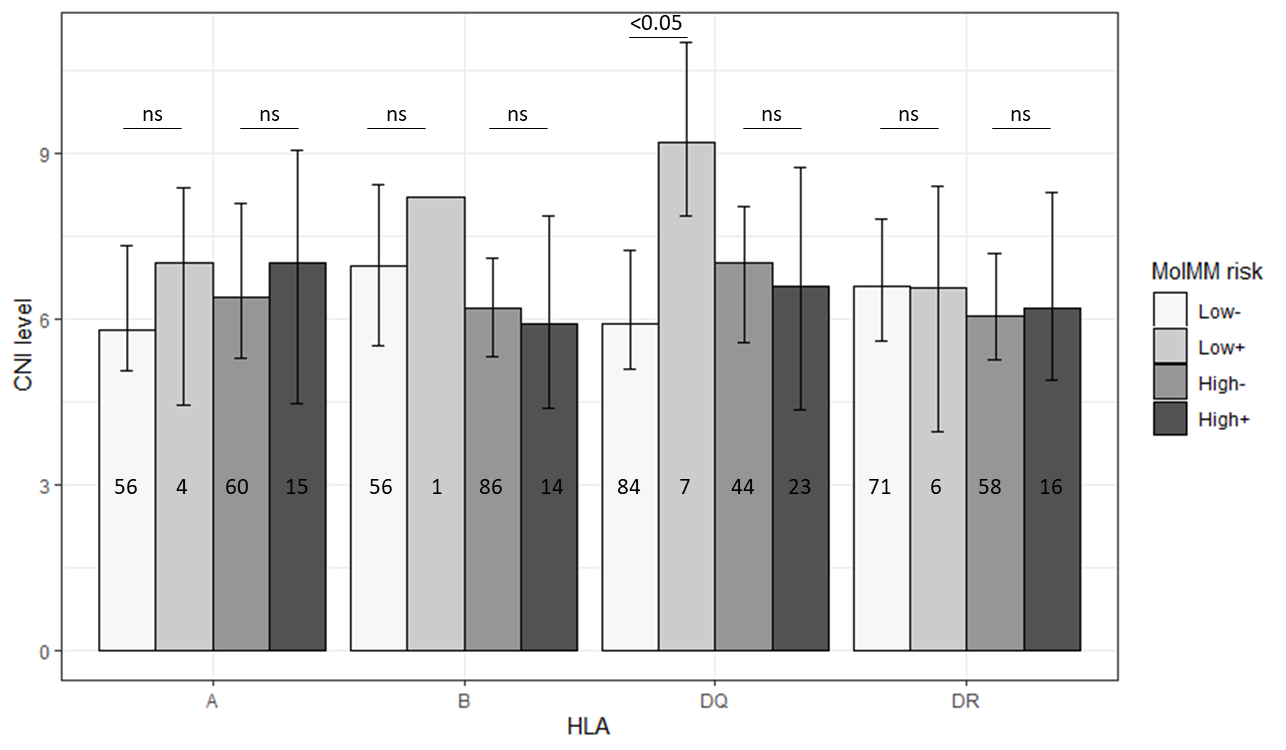


**Figure S3:** Comparison of CNI levels in patients with and without DSA in each molecular risk category (EMS3D): A) Percentage of times CNI were >5 ng/ml prior to DSA formation; B) CNI levels in the 6 months preceding DSA formation. DSA positive (+) versus negative (-) patients were compared using the Mann Whitney test. For DSA- patients, CNI levels were taken from the whole follow-up period. Results are presented as median ± inter-quartile range. Numbers represent number of patients in each category. DSA+ patients within 6 months of transplant were excluded from analysis (A). ns=not significant


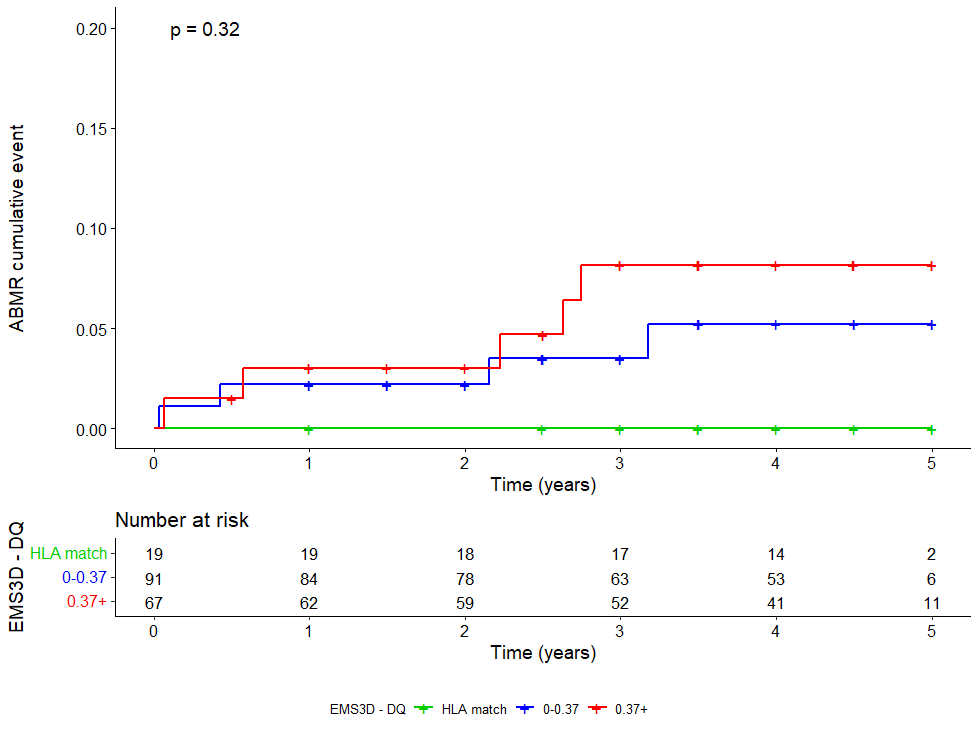


**Figure S4:** Cumulative event of ABMR using EMS3D-DQ risk categories. Comparison between groups was performed using log-rank analysis.
